# The development of a global chiropractic rehabilitation competency framework by the World Federation of Chiropractic

**DOI:** 10.1186/s12998-019-0249-8

**Published:** 2019-05-29

**Authors:** Pierre Côté, Deborah Sutton, Richard Nicol, Richard Brown, Silvano Mior

**Affiliations:** 1 Canada Research Chair in Disability Prevention and Rehabilitation, University of Ontario Institute of Technology (UOIT), 2000 Simcoe Street North, Oshawa, Ontario L1H 7L7 Canada; 2 UOIT-CMCC Centre for Disability Prevention and Rehabilitation, University of Ontario Institute of Technology (UOIT) and Canadian Memorial Chiropractic College (CMCC), 6100 Leslie Street, Toronto, Ontario M2H 3J1 Canada; 3 Faculty of Health Sciences, University of Ontario Institute of Technology (UOIT), 2000 Simcoe Street North, Oshawa, Ontario L1G 0C5 Canada; 4 World Federation of Chiropractic, Disability and Rehabilitation Committee, 160 Eglinton Avenue East, Suite 601, Toronto, Ontario M4P 3B5 Canada; 5 Canadian Memorial Chiropractic College, 6100 Leslie Street, Toronto, Ontario M2H 3J1 Canada

**Keywords:** Chiropractic, Competency-based education, Rehabilitation, Disability, Global Health, Health promotion

## Abstract

The World Health Organization (WHO), in its “Rehabilitation 2030 A Call for Action”, identified the need to strengthen rehabilitation in health systems to meet the growing demands of current and future populations. Greater access to rehabilitation services is required to secure the achievement of the United Nation’s third Sustainable Development Goal, “Ensure healthy lives and promote well-being for all at all ages”. To support this mandate, WHO issued a call for non-governmental organizations, associations and institutions to share their rehabilitation-related competency frameworks which will be used to construct a global rehabilitation competency framework. In response to this call, the World Federation of Chiropractic (WFC) developed a chiropractic rehabilitation competency framework. In this article, we present the chiropractic rehabilitation competency framework that will contribute to the development of the global framework in support of WHO’s strategic planning for rehabilitation. The goal of WHO’s strategic planning is to improve the integration and support of multi-disciplinary rehabilitation and establishing opportunities for global networks and partnerships in rehabilitation.

**Electronic supplementary material:**

The online version of this article (10.1186/s12998-019-0249-8) contains supplementary material, which is available to authorized users.

## Background

An increasing unmet need for rehabilitation exists globally as a consequence of the rising prevalence of non-communicable diseases and injuries and the ageing population [1]. While health plays a role in all 17 of the United Nations’ Sustainable Development Goals (SDG), the provision of rehabilitation services is necessary for the achievement of the third SDG “Ensure healthy lives and promote well-being for all at all ages” [1]. However, there are significant concerns about the ability to access adequate rehabilitation services in several regions of the world.

In February 2017, the World Health Organization (WHO) launched its “Rehabilitation 2030 A Call for Action” to: “1) draw attention to the increasing needs for rehabilitation; 2) highlight the role of rehabilitation in achieving the SDGs; 3) and call for coordinated and concerted global action towards strengthening rehabilitation in health systems” [1]. Later in August 2018, WHO issued a call for non-governmental organizations, associations and institutions to share their rehabilitation-related competency frameworks. These submissions will inform development of a global rehabilitation competency framework to improve the integration and support of multi-disciplinary rehabilitation and establish opportunities for global networks and partnerships in rehabilitation.

## Objective

The objective of this article is to present a chiropractic rehabilitation competency framework developed by the World Federation of Chiropractic Disability and Rehabilitation Committee (WFC DRC). The framework includes a set of core competencies: communication, knowledge, technical skills, clinical reasoning, values, and reflection which taken together will ensure the rights, well-being and safety of patients are protected and that chiropractors will employ evidence-informed practices in the delivery of patient care [2, 3].

## Methods

### Development of the framework

The Secretary-General of the World Federation of Chiropractic (WFC) invited the Chair of the WFC DRC to respond to the WHO call for rehabilitation competency frameworks. The WFC DRC Chair formed a subcommittee of individuals representing expertise in rehabilitation and disability, and educational frameworks for a competency-based education for chiropractors, to collaboratively develop the rehabilitation competency framework.

The WFC DRC subcommittee reviewed WHO documents and other key resources from chiropractic councils and educational institutions to inform the development of the chiropractic rehabilitation competency framework (see Additional files 1 and 2). The subcommittee developed the competency framework guided by the internationally accepted frameworks produced by the World Health Organization [4, 5].

The competency framework includes three key domains that groups related competencies. The first domain encompasses an overall understanding of the concepts of rehabilitation and disability, which are necessary for chiropractors to understand a person’s health and rehabilitation needs. The second domain focuses on protection of the public and provision of rehabilitation services within the boundaries of laws, policies and regulations, and ensures the highest quality and professional standard of care. The third domain describes the competencies that a chiropractor requires in the rehabilitation management of disability and other health conditions. The competencies within each domain were selected to represent the essential knowledge, skills, attitudes and behaviors to be demonstrated by chiropractors providing rehabilitation services [6].

The subcommittee met on six occasions to develop the chiropractic rehabilitation competency framework. Each of the three key domains and associated competencies were discussed and independently reviewed by each member of the subcommittee. A draft framework was approved by the subcommittee through consensus. The final draft framework was then submitted to the WFC-DRC for critical review in September 2018 and comments were integrated in the final framework. WFC-DRC members represent various world regions (Africa (*n* = 1), Australia (*n* = 1), Europe (*n* = 7), North American (*n* = 9), South American (*n* = 1)) and professions (physical therapy (*n* = 3), psychology (*n* = 1), medicine (*n* = 1), chiropractic (*n* = 13), occupational therapy (*n* = 1)). An external chiropractic representative from Asia also provided peer review of the draft document in September 2018, to ensure representation across all world regions. Finally, the Chair of the WFC-DRC submitted the final framework to the WFC Secretary-General who submitted it to the WFC council members. WFC council members reviewed the final framework and approved it through consensus. The final framework was submitted to WHO on September 30, 2018.

### Domains and competencies

The framework is based upon a universal understanding of the concepts of rehabilitation, disability and person-centered care (Table 1).

**Table 1:** Definitions of rehabilitation, disability and person-centered care

Rehabilitation	A “set of interventions designed to reduce disability and optimize functioning in individuals with health conditions in interaction with their environment” [1]. Health conditions may refer to disease, disorder, injury or trauma, but may also include conditions such as pregnancy, ageing, stress, congenital anomalies or genetic predisposition [1]. Rehabilitation services may be accessed by people living with all types of health conditions, and is not limited to those who experience a disability [1].
Disability	The World Health Organization (WHO) follows the International Classification of Functioning, Disability and Health framework in defining disability as an “umbrella term for impairments, activity limitations and participation restrictions” [7]. Disability is the interaction of body function and structure, activity and participation in the context of environmental and personal factors upon an existing health condition [8]. Disability is a human rights issue encompassing stigmatization, discrimination and inequalities [7]. Individuals with disabilities continue to experience separation and/or segregation and exclusion in many communities around the world despite the work to shift towards community, educational and work inclusion. Persons with disability include people who are traditionally understood as disabled, such as persons who are wheelchair users, those who are blind or deaf, people with intellectual impairments or mental health conditions. It also includes the wider group of persons who experience difficulties in functioning due to a wide range of conditions such as non-communicable diseases, infectious diseases, neurological disorders, injuries and conditions that result from the aging process [7].
People-centered care	People-centered health services consciously consider perspectives of individuals, families and communities, and recognizes each as participants and beneficiaries of such services, while respecting their needs and preferences in a humane way. In so doing, people are educated and informed in order to be active participants in their care. People-centered care focuses on the holistic health needs and expectations of people rather than their disease [9]. (http://www.who.int/servicedeliverysafety/areas/people-centred-care/global-strategy/en/).

The competency framework is presented as a tabular matrix stratified by the three domains and their related competencies (Table 2). Each competency was further defined as measurable standards related to knowledge and skills that establish the expected level of performance (Table 3). Due to the interrelated nature of standards of attitudes and behaviors that cross all three domains, they are summarized in a separate table (Table 4). Certain competencies may not apply to all settings or environments. Therefore, our recommendations need to be interpreted and applied while respecting the defined roles and responsibilities of chiropractors in the specific region.

**Table 2:** Domains and competencies for rehabilitation of disability in chiropractic

Domains		Competencies	
Domain 1.	Basic concepts of rehabilitation and disability	Competency 1.1	Demonstrate an understanding of the contemporary frameworks of rehabilitation and disability
		Competency 1.2	Effectively communicate with patients and all/key stakeholders involved in the rehabilitation process
Domain 2.	Legal, regulatory and ethical components	Competency 2.1	Apply in clinical practice the laws, regulations and policies that impact delivery of rehabilitation services
		Competency 2.2	Apply in clinical practice the ethical delivery of services
		Competency 2.3	Deliver rehabilitation services in line with regulatory standards
		Competency 2.4	Advocate for rehabilitation services as a means of achieving optimal outcomes, including return to work
Domain 3.	Rehabilitation management of disability and other health conditions	Competency 3.1	Clinical assessment—evaluates patients’ health status and related circumstances to develop a working diagnosis
		Competency 3.2	Formulate a clinical plan of management
		Competency 3.3	Monitor and evaluate progress of care and health outcomes
		Competency 3.4	Participate in implementing the rehabilitation plan within the unique cultural and environmental context

**Table 3:** Core competencies and associated knowledge and skills

Domain 1	Basic concepts of disability and rehabilitation
Competency 1.1 Demonstrate an understanding of the contemporary frameworks of rehabilitation and disability	Knowledge
	• Describe the concept of rehabilitation
	• Describe the concept of disability (e.g. International Classification of Functioning, Disability and Health (ICF))
	• Identify the determinants of health within populations served
	• Explain the epidemiology of neuromusculoskeletal diseases and the factors that might prevent or delay their onset, and prognosis
	Skills
	• Diagnose and care for patients within a biopsychosocial model
	• Apply contemporary concepts of rehabilitation and disability in the implementation of services
Competency 1.2 Effective communication with patients and all/key stakeholders involved in rehabilitation services	Knowledge
	• Recognize good communication is a core skill that fosters relationships and improves outcomes
	• Understand that disability encompasses physical, mental and sensory components
	• Understand the power of words in communication
	Skills
	• Develop knowledge, attitudes and skills of effective communication
	• Recognize and interpret verbal and non-verbal communication
	• Utilize available information and communication technologies
	• Share resources and knowledge
	• Facilitate a structured, evidence-based rehabilitation encounter
Domain 2	Legal, regulatory and ethical components
Competency 2.1 Apply in clinical practice the laws, regulations and policies that impact delivery of rehabilitation services	Knowledge
	• Comprehend legal duties and responsibilities relating to rehabilitation and disability
	• Describe the basic regulations and policies of stakeholder organizations relating to rehabilitation, disability and return to regular activities
	• Explain the application of the professional duty of candour
	• Distinguish the professional and ethical boundaries expected of the chiropractor-patient relationship
	• Summarize the principles of risk management, safe practice and duty of care
	• Explain medico-legal processes and procedures, including vicarious liability
	• Describe the model of health care service provision across the different levels and sites of care at the local, regional and national level
	Skills
	• Competent acquisition of informed consent from the patient or substitute decision maker
	• Integrate and respect the diversity of patients’ cultural and religious values and belief systems
	• Demonstrate respect for individual preferences and expressed needs
	• Recognize and manage threats to safety of people requiring rehabilitation
	• Operate a safe and accessible practice environment
	• Manage health risks and public health issues, including reporting
	• Practice in accordance with laws and principles of confidentiality
	• Work with a community or population to identify the determinants of health that affect them
	• Enable and support integration of chiropractic services within the health care system context
Competency 2.2 Apply in clinical practice the ethical delivery of services	Knowledge
	• Recognize persons entitlement to enjoyment of universal and indivisible rights and freedoms
	• Describe, promote and protect human rights and freedoms of persons
	• Recognize individual involvement in decision-making
	Skills
	• Engage persons full participation in community, work and home environments
	• Engage persons active involvement in decision-making
	• Provide an accessible environment for rehabilitation services
	• Communicate clinical information with equipoise
	• Refer patients in need of accessible information about mobility aids, devices and assistive technology to appropriate providers
	• Evaluate and work within provider’s individual capacity of knowledge and skills
	• Develop rapport and trust with patients, and other stakeholders, colleagues and professionals, in compliance with privacy legislation
	• Apply ethical principles of autonomy, beneficence, non-malfeasance and justice
Competency 2.3 Deliver rehabilitation services in line with regulatory standards	Knowledge
	• Describe and apply evidence-based clinical guidelines and protocols required for clinical practice
	• Recognize and respect scope of practice of other health care professionals
	• Recognize and respect the policies and regulations governing key stakeholders
	• Acquire and incorporate advances in knowledge into clinical practice
	• Comprehend duties and responsibilities arising from statutory regulation
	• Describe the structure of the chiropractic profession and the purpose of representative bodies (including trade unions, chiropractic associations, colleges, societies and patients’ associations)
	Skills
	• Complies with professional standards and legal requirements for creation, maintenance, storage and disposal of patient records and business documents
	• Allocate finite healthcare resources appropriately
	• Apply evidence-based and management processes to achieve cost-appropriate care
	• Work collaboratively to provide an interdisciplinary approach to care
	• Collaborate with community organizations, families, employers, and other stakeholders as required
	• Critically evaluate quantitative and qualitative research to inform evidence-based practice
	• Recuse self from practice when personal health and well-being makes it unsafe to do so
Competency 2.4 Advocate for rehabilitation services as a means of achieving optimal outcomes, including return to work	Knowledge
	• Define available rehabilitation services in individualized care plans.
	• Define key stakeholders that would facilitate return to activity and work
	Skills
	• Communicate with stakeholders to facilitate return to activity and work
	• Engage stakeholders to improve rehabilitation services
	• Employ people centered care to support equitable and accessible rehabilitation services
Domain 3	Rehabilitation management of disability and other health conditions
Competency 3.1 Clinical assessment—evaluate patients’ health status and related circumstances to develop a working diagnosis	Knowledge
	• Describe normal structure and function of the human body
	• Identify and consider biopsychosocial determinants of health
	• Describe etiology, pathology, symptomatology, natural history and prognosis of neuromusculoskeletal complaints, pain syndromes and associated disorders
	• Interpret diagnostic procedures, including reliability, validity, uses and limitations
	• Identify and consider lifestyle, occupation or external factors that may influence health or well-being and return to activities/work
	Skills
	• Conduct a patient history and physical examination using knowledge of pathophysiology, basic and clinical sciences
	• Identify the need for and availability of external health records
	• Perform and/or refer for diagnostic studies when indicated, e.g. diagnostic imaging, ultrasound, clinical laboratory tests
	• Interpret diagnostic study results
	• Explain outcomes and implications, using an appropriate comprehension level, of assessment, including working diagnosis and plan of management, to the patient, family and/or caregiver
	• Formulate a differential diagnosis, where appropriate
Competency 3.2 Formulate a clinical plan of management	Knowledge
	• Recognize need for emergency care, referral and/or collaborative care
	• Identify acute, subacute and chronic phases of conditions
	• Recognize indicators of persons psychosocial, environmental, and health behavioral factors that may be comorbid with neuromusculoskeletal conditions or associated with the risk of developing other physical and/or psychological conditions
	• Knowledge and understanding of evidence-based people-centered plan of management
	• Summarizes principles of health promotion and disease prevention
	• Critical appraisal, translation and application of health care knowledge
	Skills
	• Develop therapeutic goals and prognoses based upon individual needs and strengths
	• Provide advice, explanations and reassurance related to working diagnosis and clinical plan of management
	• Employ evidence-based rehabilitation interventions for the purpose of optimizing function
	• Counsel patient, family and/or caregiver on preventive, supportive, concurrent and referral care
	• Engage and support patient, family, caregiver, employer, union and/or community stakeholders in shared decision-making of a plan of care, including identifying risks, benefits, natural history, alternative care and patient preferences
Competency 3.3 Monitors and evaluates progress of care and health outcomes	Knowledge
	• Describe and employ reliable and valid outcome measures
	• Identify patient expectations, experience and patient-reported outcome measures
	• Recognize indications for cessation or referral of care based upon patient outcomes
	Skills
	• Monitor patient clinical status and modify diagnosis and care plan as indicated
	• Modify plan of care, including consideration of alternative options, based upon individual’s progress and external factors
	• Manage potential complications or adverse events arising from plan of care and have procedures in place to manage these circumstances effectively
	• Discharge from care based upon mutually agreed upon goals
Competency 3.4 Participates in implementing the rehabilitation plan within the unique cultural and environmental context	Knowledge
	• Explain the relevance of a history and assessment, taking into account home, school, work and community environments
	• Describe the environment and culture of the community
	• Recognize responsibility to protect, promote and advance the health and well-being of individuals, communities and populations
	• Discuss with stakeholders to facilitate optimal recovery, timely return to work (where appropriate) and restoration of agreed quality of life parameters
	Skills
	• Support patient participation and inclusion in all aspects of the home, school, work and community environments
	• Deliver rehabilitation services that are congruent with the local cultural, sociodemographic, work and environmental context

**Table 4:** Attitudes and behaviors that are an essential component of competencies

• Treat persons with respect for her/his human rights and dignity	
• Respect person’s autonomy, independence and preferences including their right to consent or refuse assessment and treatment in shared decision-making	
• Treat persons in a non-judgmental and non-discriminatory manner	
• Embark upon rehabilitation services as a continuous participatory process, to reach mutually agreed upon outcomes	
• Demonstrate empathy	
• Respect and value diversity and differences at individual and cultural levels	
• Listen to others with respect and use non-authoritarian communication	
• Respect confidentiality and privacy in the provision of rehabilitation services	
• Adhere to professional ethical standards	
• Demonstrate awareness of one’s own attitudes, values and beliefs that may impact the ability to provide confidential, non-discriminatory, non-judgmental and respectful care	
• Respect accessibility, needs and requirements of persons	

### Domain 1: basic concepts of rehabilitation and disability

Chiropractors require a foundational understanding of rehabilitation and disability (Tables 1, 2 and 3). Disability is universal, yet diverse. It is neither simply a biological, psychological or social phenomenon. It is an umbrella term for impairments, activity limitations and participation restrictions [7]. Chiropractors require specialized skills in interpersonal communication, communication methods, technology and rehabilitation. Chiropractic rehabilitation competencies are framed upon the basic premise that culturally appropriate people-centered care is at the core of all patient/provider interactions (Table 1).

### Domain 2: legal, regulatory and ethical components

This domain addresses social determinants of health in the context of statutory, regulatory and ethical frameworks guiding the delivery of rehabilitation services (Tables 2 and 3). It recognizes the contribution of multiple stakeholders, including healthcare professionals, policy makers and community representatives to deliver concerted, coordinated services in a person-centered environment [10].

Chiropractors should be aware of the legal and ethical issues and principles relating to the delivery of rehabilitation services. Human rights, including privacy and confidentiality, ethical issues around governance and management of facilities and understanding of competing interests are all components that are key elements of this domain [11].

### Domain 3: rehabilitation management of disability and other health conditions

The objective of rehabilitation is to optimize function, enabling individuals to be as independent as possible, to participate in education, to be economically productive and fulfill meaningful life roles [1]. (Tables 2 and 3) It is essential that rehabilitation be offered in both health care facilities (clinics and hospitals) and community settings to ensure timely access to services along the continuum of care and across the lifespan [12]. The chiropractor should engage the patient to ascertain their valued roles within the context of environmental and personal factors. Restoration of body functions and structure are not enough alone to address the effect of disability, which must be framed within a biopsychosocial model. Rehabilitation should have both a beginning and an end. A stage of equilibrium in which active interventions are no longer required may occur. However, this does not preclude the need for future rehabilitation services to further a person’s participation in education, employment and the community [13].

Attitudes, knowledge and skills are required to demonstrate core competencies.

Embodied in the provision of rehabilitation services are respect for human rights, principles of equity, meaningful participation and inclusion. Accordingly, there are underlying attitudes and behaviors that are an essential component of competencies and interrelated to each of the identified domains (see Table 4).

## Discussion

The rising prevalence of non-communicable diseases and injuries, along with an ageing population has contributed to a growing demand for rehabilitation worldwide [1]. However, the capacity to provide rehabilitation is limited or non-existent in many areas of the world, thus failing to meet population needs. Rehabilitation serves to optimize function and support those with health conditions to remain as independent as possible, participate in education, be economically productive and fulfill meaningful roles. Consequently, rehabilitation plays a foundational role in achieving the third SDG, “Ensure healthy lives and promote well-being for all at all ages” [14]. The objective of this commentary was to describe the development of a chiropractic rehabilitation competency framework.

The WFC DRC developed a chiropractic rehabilitation competency framework as a reference document to inform the development of the WHO global rehabilitation competency framework. Our framework includes three domains, their related core competencies and associated attitudes, knowledge and skills. Each domain is comprised of core competencies with a synopsis of the attitudes, knowledge and skills required to demonstrate these core competencies. The chiropractic rehabilitation competency framework will contribute to the development of the WHO global rehabilitation competency framework to support workforce evaluation and strategic planning for rehabilitation globally.

We surveyed and used current WHO documents and key resources from chiropractic councils and educational institutions to develop and structure the framework (see Additional file 1). The selected resources were considered to be most salient to the identification of domains and core competencies which are essential to chiropractic practice. Nevertheless, it is possible that we missed material that would have further informed the framework. We conducted a qualitative synthesis of the knowledge presented in retrieved documents, but we did not critically appraise the content of these documents. However, the critical review of the draft framework by the subcommittee, the WFC DRC and the WFC council allowed for a broad range of individual opinions and knowledge to be considered throughout the iterative development process.

## Conclusion

Overall, we have presented a chiropractic rehabilitation framework consisting of three domains: basic concepts of rehabilitation and disability; legal, regulatory and ethical components; and, rehabilitation management of disability and other health conditions. Each domain is comprised of core competencies with a synopsis of the attitudes, knowledge and skills required to demonstrate these core competencies. The chiropractic rehabilitation framework developed by the WFC DRC will help inform WHO about the contribution of chiropractic to the agenda outlined by “Rehabilitation 2030: A Call for Action”. The framework also provides a guide for chiropractic educational institutions and national associations to develop rehabilitation policies within their jurisdictions.

## Additional files

Additional file 1: Key Resources. WHO documents and key resources from chiropractic councils and educational institutions. (DOCX 21 kb)

Additional file 2: Glossary. Alphabetical list and meanings of key terms used in chiropractic rehabilitation framework. (DOCX 22 kb)
