# Supplementary material for: The development of a global chiropractic rehabilitation competency framework by the World Federation of Chiropractic
Source: Chiropr Man Therap. 2019 May 29;27:29. doi: 10.1186/s12998-019-0249-8 (PMC6540412; doi:10.1186/s12998-019-0249-8)
Supplement: Supplementary file 1 — Key Resources. WHO documents and key resources from chiropractic councils and educational institutions. (DOCX 21 kb) [file 12998_2019_249_MOESM1_ESM.docx]

Additional file 1: Key Resources

The WFC DRC subcommittee engaged in a review of WHO documents and key resources from chiropractic councils and educational institutions globally that informed the development of the chiropractic rehabilitation competency framework.

The following resources were identified and reviewed to inform the competency framework:

1. Convention on the Rights of Persons with Disabilities an Optional Protocol. New York, United Nations, 2006. (<http://www.un.org/disabilities/documents/convention/convoptprot-e.pdf>)
2. Global Health Ethics: Key Issues. Geneva, WHO, 2015. (<http://www.who.int/ethics/publications/global-health-ethics/en/>)
3. Core Competencies in Adolescent Health and Development for Primary Care Providers. Geneva, WHO, 2015. (<http://www.who.int/maternal_child_adolescent/documents/core_competencies/en/>)
4. WHO Competency Framework for Health Workers’ Education and Training on Antimicrobial Resistance. Geneva, WHO, 2018. (<http://www.who.int/hrh/resources/WHO-HIS-HWF-AMR-2018.1/en/>)
5. World Health Organization. Rehabilitation 2030: A Call for Action. Geneva, WHO, 2017. (<http://www.who.int/disabilities/care/rehab-2030/en/>)
6. Accreditation Procedures and Standards in First Qualification Chiropractic Education and Training, May 2018. European Council on Chiropractic Education. (<http://www.cce-europe.com/downloads.html>)
7. CCE Accreditation Standards, Principles, Processes and Requirements for Accreditation, January 2018. The Council on Chiropractic Education. (<http://www.cce-usa.org/uploads/1/0/6/5/106500339/2018_cce_accreditation_standards.pdf>)
8. Education Standards, Criteria for Chiropractic Programme Content and Structure, September 2017, General Chiropractic Council (GCC). (<https://www.gcc-uk.org/education/education-standards/>)
9. Exam Content, Candidate Information, March 2016. Canadian Chiropractic Examining Board. (<http://www.cceb.ca/docs/Exam-Content-CCEB.pdf>)
10. Standards for Accreditation of Doctor of Chiropractic Programmes, November 26, 2011. Canadian Federation of Chiropractic Regulatory and Educational Accrediting Boards (Federation). ( <http://www.chirofed.ca/english/pdf/Standards-for-Accreditation-of-Doctor-of-Chiropractic-Programmes.pdf>)
11. The International Framework for Chiropractic Education and Accreditation, Programme Standards, Competencies and Accreditation Policies and Procedures, June 2016. Councils on Chiropractic Education International. (<https://www.cceintl.org/important-documents>)
