# Supplementary material for: The development of a global chiropractic rehabilitation competency framework by the World Federation of Chiropractic
Source: Chiropr Man Therap. 2019 May 29;27:29. doi: 10.1186/s12998-019-0249-8 (PMC6540412; doi:10.1186/s12998-019-0249-8)
Supplement: Supplementary file 2 — Glossary. Alphabetical list and meanings of key terms used in chiropractic rehabilitation framework. (DOCX 22 kb) [file 12998_2019_249_MOESM2_ESM.docx]

Additional File 2: Glossary

Attitude—A person’s views (values and beliefs) about a thing, process or person, which influence behavior.^1^

Behaviour—A person’s way of relating or responding to the actions of others or to an environmental stimulus.^1^

Candour-Healthcare professionals have a duty to be open and honest with patients when something goes wrong with their treatment or care causes, or has the potential to cause harm or distress. Moreover, healthcare professionals have a duty to be open and honest with colleagues, employers and relevant organizations, and take part in reviews and investigations upon request. Health care professionals need to be open and honest with regulatory bodies, raising concerns when appropriate. (<https://www.nmc.org.uk/standards/guidance/the-professional-duty-of-candour/read-the-professional-duty-of-candour/>)

Chiropractor—A healthcare profession concerned with the diagnosis, treatment and prevention of disorders of the neuromusculoskeletal system and the effects of these disorders on general health. There is an emphasis on manual techniques, including joint adjustment and/or manipulation.^2^

Competency—Sufficient knowledge and psychomotor, communication and decision-making skills and the attitudes to enable the performance of actions and specific tasks to a defined level of proficiency.^1^

Core competency—A competency that a sector (e.g. health, education) has agreed is essential for a person to perform requisite functions and tasks. In health education core, or essential, competencies are the aspects of a subject or discipline that are common to all students, essential to practice, and essential to master in order to graduate from an academic program and enter into professional practice.^1^

Evidence-based practice—“The conscientious, explicit and judicious use of current best evidence in making decisions about the care of the individual patient. It means integrating individual clinical expertise with the best available clinical evidence from systematic research”.^3^

Knowledge—An individual’s understanding of a subject, including not only facts and information but also the ability to apply them for a specific purpose.^1^

Skill—Ability learned through pre-service and continuous professional education and/or acquired through experience to perform specific actions or tasks to a specified level of measurable performance.^1^

References

1. World Health Organization. *Core Competencies in Adolescent Health and Development for Primary Care Providers*. Geneva: WHO, 2015.

2. World Health Organization. WHO Guidelines on Basic Training and Safety in Chiropractic. Geneva, Switzerland. 2005.

3. Sackett DL, Rosenberg WM, Gray JA, Haynes RB and Richardson WS. Evidence based medicine: what it is and what it isn't. 1996. *Clin Orthop Relat Res*. 2007; 455: 3-5.
